# Low STR variability in the threatened marsh deer, *Blastocerus dichotomus*, detected through amplicon sequencing in non-invasive samples

**DOI:** 10.1590/1678-4685-GMB-2022-0105

**Published:** 2022-10-24

**Authors:** Laura Irene Wolfenson, Gregory R. McCracken, Daniel E. Ruzzante, Patricia Mirol, Antonio Solé-Cava

**Affiliations:** 1 Museo Argentino de Ciencias Naturales, “Bernardino Rivadavia”, División de Mastozoología, Ciudad Autónoma de Buenos Aires, Argentina.; 2 Dalhousie University, Department of Biology, Halifax, Nova Scotia, Canada.; 3 Universidade Federal do Rio de Janeiro, Instituto de Biologia, Departamento de Genetica, Centro Nacional para a Identificação Molecular do Pescado (CENIMP), Rio de Janeiro, RJ, Brazil.

**Keywords:** Next Generation Sequencing, mammal, cervid, microsatellites

## Abstract

*Blastocerus dichotomus* is the largest deer in South America. We have used 25 microsatellite markers detected and genotyped by Next Generation Sequencing to estimate the genetic variability of *B. dichotomus* in Argentina, where most of its populations are threatened*.* Primer design was based on the sequence of a shallow partial genome (15,967,456 reads; 16.66% genome coverage, mean depth 1.64) of a single individual. From the thousands of microsatellite *loci* found, even under high stringency selection, we chose and tested a set of 80 markers on 30 DNA samples extracted from tissue and feces from three Argentinean populations. Heterozygosity levels were low across all *loci* in all populations (H=0.31 to 0.40). Amplicon sequencing is a fast, easy, and affordable technique that can be very useful for the characterization of microsatellite marker sets for the conservation genetics of non-model organisms. This work is also one of the first ones to use amplicon sequencing in non-invasive samples and represents an important development for the study of threatened species.

*Blastocerus dichotomus* is the largest deer in South America, that inhabits all types of wetlands, such as flooded grasslands, lagoons and swamps with floating marshes (Duarte and González, 2010), which gives it the common name of “marsh deer”. It is listed as Vulnerable by the IUCN. In Argentina, most of its populations are threatened (Pereira et al., 2019) which is why starting to address its situation from a genetic perspective is imperative.

Microsatellite *loci* are widely distributed in the genomes of eukaryotes and have been used without major difficulties in many species (for example, Dayon et al., 2020). The development of markers with the modern Next Generation Sequencing (NGS) approach, which involves the partial sequencing of a genome, makes the analysis of thousands of microsatellite *loci* possible, allowing them to be chosen under much more stringent conditions. Moreover, NGS technologies can also be used to genotype microsatellites, through PCR amplicon sequencing, which allows for a faster, easier, and more affordable process (Andrews et al., 2018). This approach helps to overcome some of the limitations of former genotype-by-fragment-size microsatellite analyses: unambiguous allele identification, additional information besides number of repeats, and possibility of comparing genotypes across different laboratories (Andrews *et al.*, 2018). Also, the application of NGS techniques for both the design and genotyping of microsatellites allows the analyses of short target sequences (from 40 / 50 bp), which can be particularly important when analyzing low quality or degraded DNA.

The objective of this study was to estimate the genetic variability of the marsh deer in Argentina using a considerable number of new microsatellite markers*.* We used NGS techniques for the development and characterization of the markers and estimated genetic variability in tissues and non-invasive samples.

We used a blood sample from a marsh deer from the Paraná River Delta for an initial genome analysis. DNA extraction was performed with a Zymo Research kit (Quick DNA Microprep kit) and sonicated with a Covaris M220 Focused-Ultrasonicator for 120 s. The resulting fragments had a mean size of 950 bp. For genomic library construction, we used the Illumina TruSeq kit, following the manufacturer’s instructions. Sequencing was done in an Illumina MiSeq, using MiSeq Reagent kit v2 2x250 paired ends following the manufacturer’s instructions. After sequencing, demultiplexing and read quality analysis were performed using the Illumina Base Space software. Ninety-eight percent of the resulting reads were used to assemble a partial genome with the software ABySS (Simpson et al., 2009). Genome coverage, understood as the percentage of the genome that had at least one nucleotide sequenced, was estimated with Sequencing Coverage Calculator (Illumina). Mean depth, estimated as the average number of times that each nucleotide was sequenced (Sims et al., 2014) was calculated as L * N / G, with L being the length of the reads, N being the total number of reads and G the length of the reference genome. Since no genome is available for the species we studied, data from the phylogenetically close white-tailed deer, *Odocoileus virginianus*, whose genome size is 2.4 GB (London et al., 2022) was used. The assembled contigs were used as an input for the microsatellite search using MSATCOMMANDER 1.0.8 MacOS version (Faircloth, 2008). We searched for di, tri and tetranucleotides with a minimum of seven, seven and five repeats respectively. We then designed the primers with the same program, using the following parameters: final product length 30-130bp, primer length 18-23bp, melting temperature between 57 °C and 65 °C, GC content 30-70%, and a GC clamp in the 3’ end. From the thousands of primer pairs found, we selected a set of 80 markers at random, that were synthetized with the addition of a sequencing tail (Left sequencing tail: CCCTACACGACGCTCTTCCGATCT, right sequencing tail: GTTCAGACGTGTGCTCTTCCGATCT). These were ordered from Macrogen Korea.

We extracted DNA from feces (N=12) with the Quick-DNA Fecal/Soil Microbe Miniprep Kit (Zymo Research) and tissues (N=18) with the salting out protocol (Miller et al., 1988), from different Argentinean populations: 19 samples from Paraná River Delta (Buenos Aires and Entre Ríos provinces), 6 from Esteros del Iberá (Corrientes province) and 5 from El Bagual Reserve (Formosa province). 

All chosen primers were screened together with the software AutoDimer (Vallone and Butler, 2004) to check for the possibility of heterodimers and hairpins, and then divided into four multiplexes of 20 primer pairs each. Amplicon libraries were built in the Marine Gene Probe Lab at Dalhousie University using the Qiagen Multiplex reagent kit, following manufacturer’s instructions. Final volume was scaled to 5 µl per reaction. The cycling program consisted of 94 °C for 15 min, 20 cycles at 94 °C for 30 s, 57 °C for 180 s, 72 °C for 60 s, and a final extension at 68 °C for 30 min. The final products were diluted with 20 µl of ultrapure water (see Zhan et al., 2017 for more details). These products were used as a template for the second PCR, which adds the indices, and consists of 2.15 µl of ultrapure water, 0.5 µl 10x buffer, 0.2 mM of each dNTP, 0.2 µM of indexed oligo, 0.3 µl of diluted PCR product and 0.25 U of TSG DNA polymerase (Bio Basic, Markham, ON, Canada), in a final volume of 5 µl per reaction. The cycling program consisted of 95 °C for 120 s, 20 cycles of 95 °C for 20 s, 60 °C for 60 s, 72 °C for 60 s, and a 72 °C final extension for 10 min (Zhan *et al.*, 2017). We screened all the PCRs, observing DNA bands by electrophoresis, using 2% agarose gels stained with gel green (Biotium, Fremont, CA, USA). Products were pooled in equal proportions and then purified using a 1.8:1 of Sera-Mag Speedbeads (GE Healthcare, Little Chalfront, UK). Library quantification was done with the Kapa Library Quantification kit (Roche, Pleasanton, California) following the manufacturer’s protocol. The library was diluted to 15 pM and sequenced on an Illumina MiSeq, using the MiSeq 150 cycle V3 single-read kit. Amplicons from feces and tissues were sequenced independently using different flow cells. This prevents reads from good quality samples overwhelming reads from samples of lower quantity and quality DNA. DNA from feces was analyzed using seven replicates each to build a consensus genotype, as recommended for this type of material (Taberlet et al., 1996). After sequencing, indexed samples were demultiplexed automatically with MiSeq Sequence Analysis software. This resulted in the creation of one FASTQ file per individual, which contained the sequences of all the corresponding microsatellites. These files were used as input for the MEGASAT software (Zhan *et al.*, 2017), which genotyped all the *loci* automatically, and generated histograms for manual visual genotyping. In addition to analyzing MEGASAT plots, we analyzed the sequences of reads of different lengths from each marker. We aligned the potential alleles with the program GENEIOUS PRIME 2020 (Kearse et al., 2012). This was done to verify the identity of the markers in relation to the original sequences used to design them, excluding markers whose polymorphism was due to indels in the flanking regions or the tandem repeat zone: a further advantage of this method compared to the genotyping by size on a capillary DNA sequencer, where size homoplasy may be a problem. 

We used MICROCHECKER 2.2 (Van Oosterhout et al., 2004) to detect large allele dropout and null alleles. In the case of non-invasive samples, we calculated the rate of false alleles and allelic dropout within replicates with GIMLET 1.3 (Valière, 2002). We calculated polymorphic information content (PIC) with the online tool Gene Calc (See the Internet Resources Section). We used Arlequin to calculate H_O_, H_E_, number of alleles per locus and to test Hardy-Weinberg and linkage disequilibria. 

The sequencing produced 15,967,456 reads. Genome coverage was 16.66%, and mean depth was 1.64. The assembly produced 1,347,182 contigs. The MSATCOMMANDER search found 12,658 microsatellites, from which 511 primer pairs were designed with the conditions described above and 80 were selected from those.

After the amplicon sequencing process, we retained 25 polymorphic markers out of the original 80 tested (Table 1). Null alleles were found for Bdi30, Bdi51, Bdi57, Bdi59 and Bdi65 *loci*. The allele dropout rate varied between 0.01 and 0.014. In the case of non-invasive replicates, the false allele rate varied between 0.01 and 0.20 and the allelic dropout rate between 0.01 and 0.14, which shows the importance of making replicates when working with this kind of samples. We calculated diversity indices for the three populations (Parana River Delta (PRD): N=19, Ho = 0.38±0.19, He=0.31±0.22; El Bagual (EB): N=5, Ho=0.38±0.24, He=0.40±0.32; Esteros del Ibera (EI): N=6, Ho=0.39±0.24, He=0.38±0.32). Detailed *loci* data are presented for the Paraná River Delta, the population with the largest N (Table 2). None of the markers showed deviations from Hardy-Weinberg equilibrium after applying the False Discovery Rate approach (Pike, 2011) to the p-values. There was no evidence for linkage disequilibrium for any pairs of *loci*. 

**Table 1 -:** Characterization of 25 polymorphic microsatellite markers on the marsh deer (*Blastocerus dichotomus*).

Locus	Left sequence	Right sequence	Repeat motif	NA	Allele size range (bp)	PIC	GeneBank accession no.
Bdi4	TATCCCACCTGCCTCTCAAC	CTGTTTCATACCACTCACCCTG	(AAAT)_n_	2	93-101	0.16	OL998317
Bdi6	CTTCTGGCAATGCAGGAGAC	TGTCAGGCAGCATTCTCTTTC	(AGAT)_n_	2	104-112	0.16	OM001701
Bdi9	CCTGGGAAGTATCTTTGGTTCC	AGTGGACAGAATGAGAGGGC	(AC)_n_	2	112-132	0.25	OM001702
Bdi10	TCAGCCCAGGATAACACAGG	GCACATGAGTATCTATGCGCG	(AC)_n_	2	79-81	0.24	OM001703
Bdi11	ACCACCTCCTTCTGTCATGG	ACATCTTTGAGCTGCGGTTC	(TG)_n_	2	127-131	0.26	OM001704
Bdi12	ACACAGAGAAGTCCAACAAGC	GGAGATTCCCATTTACGGTCC	(CT)_n_	2	114-116	0.34	OM001705
Bdi18	TCCAGTGAGTCTTCCCTGAG	GTGACTTAACCTACACGCAC	(TG)_n_	2	90-92	0.19	OM001706
Bdi19	CAGTTCATGTGGGTGTTCGC	CAGGAAGGCTGCAAGGAAAG	(TG)_n_	3	140-144	0.37	OM001707
Bdi21	TGCCCAGGTAATCAATAAGCTC	TTCACTGAGATATGAGCCCTC	(TG)_n_	3	101-107	0.36	OM021322
Bdi24	TGAGAGCTACTTTGGCCTTTG	GTTGGACACGACTGAGCAAC	(TG)_n_	5	63-35	0.36	OM021323
Bdi26	TAACCCAGGGATCGGAACTG	GACAACAAACAACAGTGGTAGC	(AC)_n_	2	118-120	0.29	OM021324
Bdi30	ACGAAGGCACCTGGTTTAAC	GCAGGGATGAGCTGACCAG	(TG)_n_	3	120-124	0.37	OM021325
Bdi36	ACCTGTGATAAACCACAGTGGG	TGGACAGCAAAGTGATTCAGTG	(AT)_n_	4	117-119	0.35	OM021326
Bdi37	ATCAGCCAGACATTGCCATC	TGAGAGCAGCTGGAATCGG	(AC)_n_	3	109-119	0.70	OM021327
Bdi39	TGGAAAGAGGAGCCTTGGAG	TGAGCAACAGTCCATTGTGTG	(AC)_n_	2	124-138	0.57	OM021328
Bdi40	AAGTGTTCCCAGCAGAAGTG	AAAGGCAGCAGGGAGAGAC	(TG)_n_	2	112-116	0.37	OM021329
Bdi42	CTGGCTTGATCAGGGTTCC	CAACATCTTGACTGTGTGGC	(TG)_n_	4	114-118	0.44	OM021330
Bdi44	GGTCTCTACTTCCTTACTAGCC	CTGGCTTGCTACAGTCCAAC	(TG)_n_	2	102-116	0.27	OM021331
Bdi49	CTCATCACATGGAAACGGCC	ACCATAGCTCTTGTTTGTCTGC	(AG)_n_	4	87-91	0.40	OM021332
Bdi51	CAAAGGAGCAAGGCACAGAG	GGAACATCCCATCATCACCC	(TG)_n_	3	83-91	0.45	OM021333
Bdi55	GCAACTGGGCACAAACTC	TGCATTTCTGGTCAAATCCC	(TG)_n_	2	105-107	0.22	OM021334
Bdi57	TGGGCTTCTACTCTTGCAGC	TGTCAGTGAAGTAATGCCTCTG	(ATT)_n_	2	113-119	0.09	OM021335
Bdi58	TGTGCTGAGTTTCTTCATGGG	CCGTATGGTGGCAGTGAAAC	(ATGT)_n_	4	96-120	0.44	OM021336
Bdi59	TACCCTTCACTCTGCTTCCC	CAGTGTCAAGTTTCTGGTTCTG	(AT)_n_	2	80-94	0.47	OM021337
Bdi65	GGACATGATTGAGCAGCTTAGG	TGGACGCCATTTCTGCTTTG	(AC)_n_	4	108-110	0.24	OM021338

NA: number of alleles, PIC: Polymorphism Information Content, Cross amp: cross-amplification.

**Table 2 -:** Diversity indices for polymorphic loci of the Paraná Delta population.

Locus	NA	H_O_/H_E_	HWE p-value
Bdi04	2	0.21/0.27	0.37
Bdi06	1	-	-
Bdi09	3	0.06/0.18	0.03
Bdi10	2	0.19/0.18	1.00
Bdi11	3	0.25/0.33	0.20
Bdi12	2	0.21/0.34	0.14
Bdi18	1	-	-
Bdi19	2	0.36/0.52	0.54
Bdi21	2	0.58/0.51	0.66
Bdi24	2	0.53/0.44	0.61
Bdi26	2	0.11/0.19	0.16
Bdi30	2	0.32/0.48	0.17
Bdi36	3	0.50/0.55	1.00
Bdi37	5	0.79/0.68	0.20
Bdi39	3	0.56/0.65	0.69
Bdi40	2	0.40/0.51	0.60
Bdi42	3	0.68/0.58	0.02
Bdi44	2	0.11/010	1.00
Bdi49	3	0.39/0.54	0.24
Bdi51	3	0.47/0.52	0.63
Bdi55	2	0.44/0.36	0.53
Bdi57	2	0.06/0.18	0.10
Bdi58	3	0.82/0.54	0.11
Bdi59	4	0.32/0.49	0.04
Bdi65	2	0.16/0.31	0.08

NA: number of alleles, HO: observed heterozygosity, HE: expected heterozygosity, HWE p-value: significative at 5% for Hardy-Weinberg equilibrium test, FIS: inbreeding coefficient, FIS p-value: significative at 5% for the inbreeding coefficient.

The use of amplicon sequencing for genotyping made it possible to affordably test a greater number of *loci* and samples than a research project that uses capillary electrophoresis genotyping reducing time and effort (for example, Latorre-Cardenas et al., 2020; Lim and Ab Majid, 2021). 

The variability of the microsatellite markers was generally low, with several *loci* having only two alleles. This is likely due to the drastic reduction in the census sizes of the populations of the species in Argentina because of hunting and habitat degradation in recent decades (Pereira et al., 2019). The observed heterozygosity results are lower than those obtained for other cervid species (Table 3), except the South Andean deer, which has slightly lower heterozygosity levels, probably resulting from very low (<2000 individuals) population size (Corti et al., 2011). Curiously, the highest reported heterozygosity (mean He=0.765) is for the Pantanal population of the same species we studied (Oliveira et al., 2009). We tried to reproduce the analyses using the same nine loci described by those authors, but most *loci* failed to amplify with our tissue and feces samples even after many rounds of optimization. The only *locus* that did amplify (Bdc65) was monomorphic in our samples. It is unclear if the differences between our results and those from Oliveira *et al.* (2009) reflect actual biological differences (related, for example, to larger populations sizes of deer populations in the Pantanal) or to technical problems related to the set of microsatellites described by them.

**Table 3 -:** Levels of microsatellite variation in some cervid species, ordered by increasing He values. He = mean Hardy-Weinberg expected heterozygosity; Ho = mean observed heterozygosity; Nal = range or mean number of alleles per locus.

Species	#loci	He	Ho	Nal	Ref
*Hippocamelus bisulcus*	14	0.344	0.341	2-3	Corti et al., 2011
*Blastocerus dichotomus*	25	0.363	0.383	2-5	This work
*Elaphurus darwinianus*	5	0.520	0.560	2-4	Zeng et al., 2007
*Capreolus pygargus*	12	0.555	0.461	4-6	Lee et al., 2015
*Cervus sichuanicus*	9	0.562	0.756	6.56	He et al., 2014
*Cervus elaphus*	14	0.600	0.580	6.8	Dellicour et al., 2011
*Ozotoceros bezoarticus*	12	0.633	0.411	7.58	Raimondi et al., 2012
*Mazama gouazoubira*	10	0.700	0.550	5.6	Caparroz et al., 2015
*Odocoileus virginianus*	11	0.750	0.721	8-20	Miller et al., 2019
*Capreolus capreolus*	8	0.760	0.590	9-18	Zachos et al., 2006
*Blastocerus dichotomus*	9	0.765	0.745	4-12	Oliveira et al., 2009

Another important result of this work was that it showed how amplicon sequencing can be very useful for the characterization and analysis of microsatellites in the Conservation Genetics of non-model organisms, since a large number of markers can be simultaneously analyzed. This can be particularly useful for organisms with low levels of heterozygosity, such as land vertebrates, whose current distribution is only about 5% or their original one (Li et al., 2015) with the consequent loss of gene variation (Willoughby et al., 2015). Over the last few years, analyses of single nucleotide polymorphisms (SNPs) began to replace microsatellites in studies of genetic variability, since they required the amplification of shorter fragments (~50bp *versus* ~100bp) (Weinman et al., 2015) and presented similar or even greater resolution to that provided by microsatellites (Weinman *et al.*, 2015). Furthermore, although microsatellite markers have been widely used in population genetic studies, they do not provide sequence information and present inconveniences such as homoplasies and null alleles (Estoup et al., 2002). However, the use of SNPs has its own disadvantages. The platforms used to genotype SNPs require expensive and specialized equipment and may not be available to every research laboratory (Andrews et al., 2018). They are also biallelic and may be worse predictors of genetic variability at the genomic level than microsatellites (Smitz et al., 2016). It has been shown that the number of SNP *loci* needed to achieve equivalent statistical power is between four and twelve times higher than for microsatellites (Liu et al., 2005). More importantly, many of the currently available SNPs methodologies (such as those from the RADSeq family; Andrews *et al.*, 2018) require genomic DNA of good quality, which is not always available in conservation genetic studies, particularly of rare or elusive species. 

This work is one of the first to use amplicon sequencing in non-invasive samples, in addition to Eriksson et al. (2020) and De Barba et al. (2017). This is particularly important considering that the low quality and quantity of the DNA from fecal samples hinders their analyses by mainstream SNP methods like RADseq, GBS or WGS (Andrews et al., 2018). 
